# Exploring the Health Effects of Phytoestrogens

**DOI:** 10.3390/metabo16060410

**Published:** 2026-06-12

**Authors:** Vladimír Kraus, Anna Birková, Miroslava Majerníková, Beáta Čižmárová

**Affiliations:** 1 Department of Gyneacology and Obstetrics, Faculty of Medicine, Pavol Jozef Šafárik University in Košice, Trieda SNP 1, 040 11 Košice, Slovakia; vladimir.kraus1@upjs.sk; 2 Department of Medical and Clinical Biochemistry, Faculty of Medicine, Pavol Jozef Šafárik University in Košice, Trieda SNP 1, 040 11 Košice, Slovakia; anna.birkova@upjs.sk (A.B.); miroslava.majernikova26@gmail.com (M.M.); 3 Gynecological Clinic, Prešov, Kováčska 15, 080 01 Prešov, Slovakia

**Keywords:** phytoestrogen, puberty disorders, human health

## Abstract

Background/Objectives: Phytoestrogens are secondary plant metabolites produced via the phenylpropanoid pathway. They include a broad spectrum of chemical compounds, such as phenolics, flavonoids, isoflavones, coumestans, lignans, and others. Their chemical structures resemble those of estradiol, and they exhibit biological effects similar to those of human estrogens, influencing many physiological processes throughout life in both men and women—including the timing and progression of puberty. Methods: The literature search included databases such as PubMed, Scopus, Web of Science, and Google Scholar with the use of specific keywords. Studies were considered eligible if they reported original findings from observational studies (cohort, case–control, and cross-sectional) or from experimental studies. Results: Phytoestrogens can modulate estrogenic activity and interact with a variety of biological pathways. These compounds may play a role in human development and pubertal processes, contribute to overall health, and potentially help alleviate menopausal symptoms and reduce the risk of certain cancers. Conclusions: Phytoestrogens have numerous positive effects on the human body across various stages of life. Their overall impact and potency, however, seem to be influenced by factors such as intake level, individual genetic variability, and the specific phytoestrogen class consumed.

## 1. Introduction

Phytoestrogens (xenoestrogens, dietary estrogens, and estrogen-like compounds) are non-steroidal compounds, secondary plant metabolites with a polyphenolic structure. These compounds have been found in over 300 plant species [1]. The term “phytoestrogen” is derived from two Greek words: “phyto,” meaning plant, and “estrogen,” meaning a hormone that affects fertility in female mammals [2]. Phytoestrogens were first observed and described in the 1940s in Western Australia, where they were associated with sheep grazing on subterranean clover (*Trifolium subterraneum*). It was believed that grazing on this crop caused hormonal imbalances in the animals, leading to various reproductive abnormalities. Manifestations included infertility, failure to conceive, maternal dystocia, and uterine prolapse in non-pregnant or infertile ewes. Pronounced mammary gland hypertrophy and excessive lactation were also observed in virgin and unbred ewes. Additionally, an increased incidence of cystic endometriosis was documented. Later research identified the cause as the estrogenic properties of isoflavones (phytoestrogens) present in the clover the sheep consumed [3]. Phytoestrogens can serve as potential mediators in plant–predator interactions, functioning as a defense mechanism where plants influence the reproductive capacity of vertebrate herbivores. By mimicking endogenous reproductive hormones, these plant-derived compounds can modulate herbivore fecundity, thereby reducing grazing pressure and plant damage [4]. Phytoestrogens could have many positive effects on the human body, such as during post-menopause and osteoporosis, and their benefits have also been observed in cancer and cardiovascular diseases. However, despite these potential positive effects, they can also produce undesirable outcomes, particularly related to reproductive physiology, by increasing susceptibility to infertility and potentially causing developmental abnormalities [5].

In our review article, we provide an overview of multifaceted molecules, including their structure, synthesis, and biotransformation in the human body. This review summarizes this group of compounds, using the most recent information from available databases.

## 2. Literature Search

**Figure 1:**
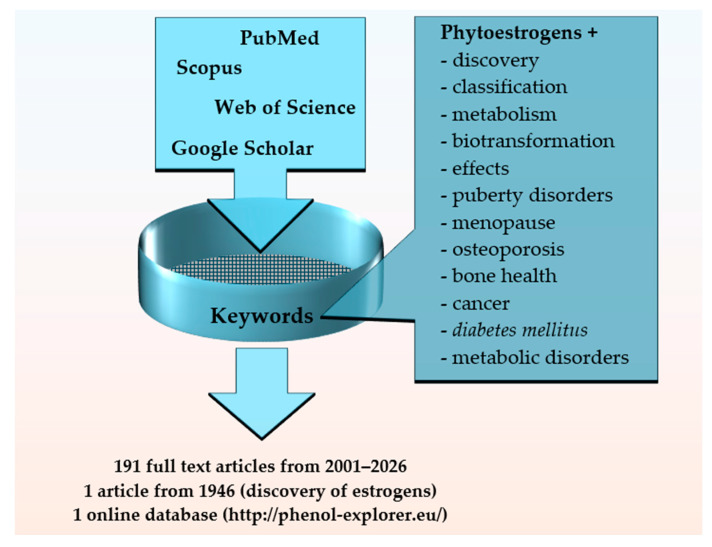
Literature search (http://phenol-explorer.eu/ (accessed on 1 March 2026)).

In our review, we provide background information on phytoestrogen exposure in humans. The literature search included databases such as PubMed, Scopus, Web of Science, and Google Scholar. For the literature search, specific keywords and their combinations were used: phytoestrogens—discovery, classification, metabolism, biotransformation, effects; puberty disorders; menopause; osteoporosis; bone health; cancer, diabetes mellitus, metabolic disorders For the preparation of this article, we used studies that reported original results from observational studies (cohort, case–control, and cross-sectional studies), book chapters, online databases, and experimental studies. We also referenced literature to offer insights into how phytoestrogens were discovered and the initial effects observed. A comprehensive analysis of the remaining research and review articles was conducted to identify the most relevant works (Figure 1).

## 3. Chemistry of Phytoestrogens

**Figure 2:**
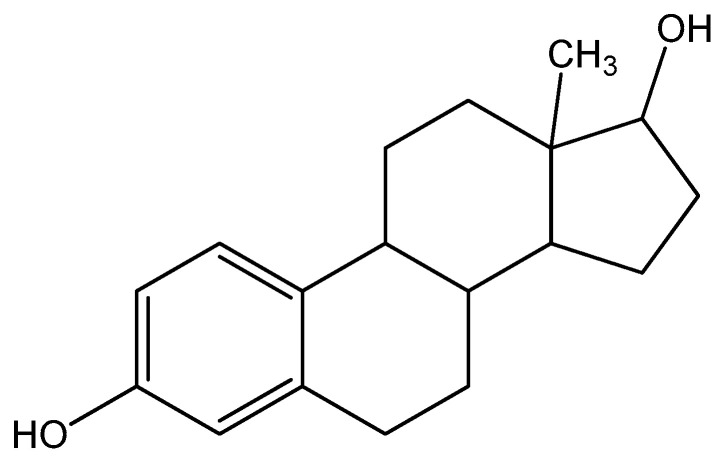
Structure of 17β-estradiol.

Phytoestrogens are non-steroidal compounds with a polyphenolic structure. These compounds display both estrogenic and antiestrogenic activities [1,2,6]. Their chemical structure closely resembles 17β-estradiol (Figure 2). This allows them to exert effects similar to those of human estrogens [2,6,7].

**Figure 3:**
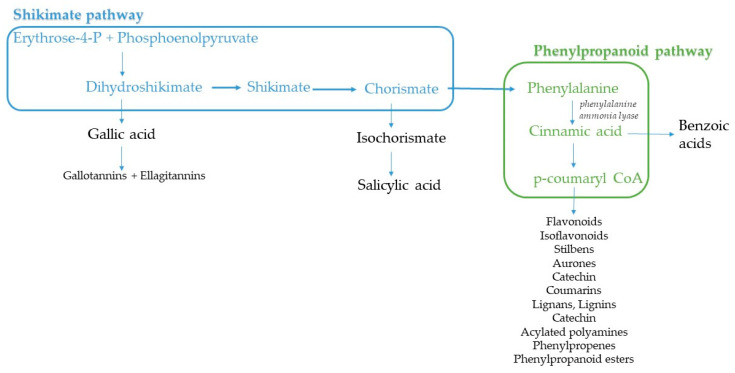
Simplified Scheme of Shikimate–Phenylpropanoid Pathway Connection (adapted from [9,10,11]).

Estrogens (17β-estradiol or estradiol, estrone, estriol, and estetrol) are hormones that play an important role in the female reproductive system and influence numerous physiological processes in both women and men [8]. Their levels fluctuate significantly across life stages, including puberty, pregnancy, and menopause or andropause [1]. Estradiol is widely regarded as the predominant and most effective estrogen in women of reproductive age; it is the main endogenous steroid hormone. Estradiol is essential for a wide range of physiological functions, including reproductive activity, metabolic regulation, mood regulation, energy homeostasis, thermoregulation, and bone health. Therefore, estrogens may offer beneficial effects during certain physiological conditions. Consequently, it seems advantageous to use phytoestrogens as hormone replacement therapy to compensate for reduced endogenous estradiol levels [4,9]. Phytoestrogens are produced via the phenylpropanoid pathway. This pathway follows the shikimate pathway, which is essential for the biosynthesis of aromatic amino acids like phenylalanine and tyrosine. Subsequently, phenylalanine serves as a starting substrate for the phenylpropanoid pathway. In this pathway, phenylalanine is converted into various plant defense molecules via the central intermediate p-coumaroyl-CoA. The key enzyme involved here is phenylalanine ammonia lyase [9,10]. This enzyme catalyzes the non-oxidative deamination of phenylalanine to trans-cinnamate and directs carbon flow from the shikimate pathway into multiple branches of the phenylpropanoid pathway [10]. The pathway produces a wide variety of defense compounds through the p-coumaroyl-CoA, including flavonoids, isoflavonoids, stilbenes, lignins, anthocyanidins, coumarins, and related molecules [11] (Figure 3).

## 4. Classification and Sources of Phytoestrogens

Phytoestrogens have a wide variety of chemical structures, which categorize them into different classes. Chemically, they are non-steroidal plant-origin compounds with a phenolic or polyphenolic structure [1]. According to the literature, there are four main classes of phytoestrogens: isoflavones, coumestans, lignans, and stilbenes [6,12,13]. However, interest in phytoestrogens has increased since their discovery, and other compounds with estrogenic or non-estrogenic properties have been identified. Table 1 summarizes the current information and expanding classification, listing other important classes of phytoestrogens, including specific examples and their food sources.

### 4.1. Biotransformation

**Figure 4:**
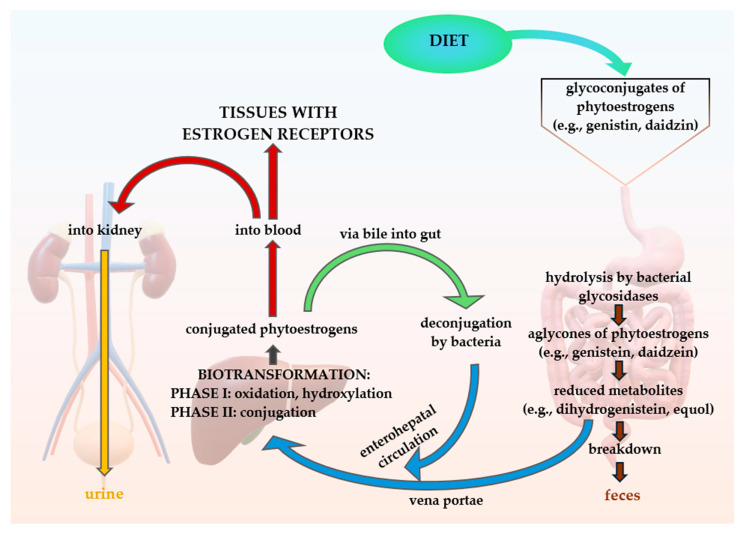
Scheme of phytoestrogens metabolism.

Phytoestrogens consumed in the diet undergo biotransformation in the body [6]. Factors influencing their bioavailability include age, gender, food matrix, dosing frequency, and their ADME properties, which encompass absorption, distribution, metabolism, and excretion [89]. Typically, the absorption and metabolism of phytoestrogens are low. The absorption rate of phytoestrogens depends mainly on their chemical structure; other influencing factors include molecule size, solubility, and the degree of glycosylation, acylation, hydroxylation, or even the degree of polymerization (Figure 4). Many phytoestrogens are glycoconjugates, so the first step involves converting them into aglycones via gut microbiota [89,90,91,92]. In the human body, phytoestrogens are metabolized in two phases, similar to how the body detoxifies drugs. In Phase I, oxidation and hydroxylation are the main processes, catalyzed by cytochrome P450 and flavin-dependent monooxygenases [90]. In Phase II, these metabolites undergo conjugation with small polar groups, facilitating their excretion through urine or bile. Glucuronidation and sulfatation, mediated by UDP-glucuronyltransferase and sulfotransferases, are the most common reactions. Conjugated phytoestrogens are excreted into bile and reenter the intestine, where bacterial deconjugation regenerates aglycones, allowing reabsorption. Free aglycones and some gut-derived metabolites can be reconjugated in the liver and intestines, increasing their solubility in body fluids. Consequently, most metabolites in human plasma are conjugated. Once in the bloodstream, phytoestrogens reach target tissues and are eventually excreted via urine or bile. Gut bacteria can also break down these metabolites, releasing free aglycones that may be reabsorbed or excreted in feces. Bacteria perform key reactions such as deglycosylation, dehydroxylation, demethylation, hydrolysis of esterified and conjugated bonds, and dehydrogenation [89,90,91,93,94].

Soy isoflavones are widely studied phytoestrogens, and pharmacokinetic studies indicate that soy isoflavones, particularly genistein and daidzein, are rapidly absorbed after oral intake, reaching peak plasma concentrations within approximately 2–9 h depending on formulation and dose [95,96,97]. Genistein shows higher systemic exposure (AUC0–∞ approximately 2000–27,500 ng·h/mL) than daidzein (approximately 1100–1200 ng·h/mL), reflecting compound-specific differences in absorption and metabolism [97,98]. Both compounds undergo extensive first-pass metabolism, with circulating exposure dominated by glucuronide and sulfate conjugates, while free aglycones remain minor components [99]. Elimination is relatively rapid and primarily urinary, with reported half-lives of approximately 3–4 h in early studies, extending to approximately 6–12 h in later reports, likely due to enterohepatic recirculation [95,100].

### 4.2. Mechanism of Action

**Figure 5:**
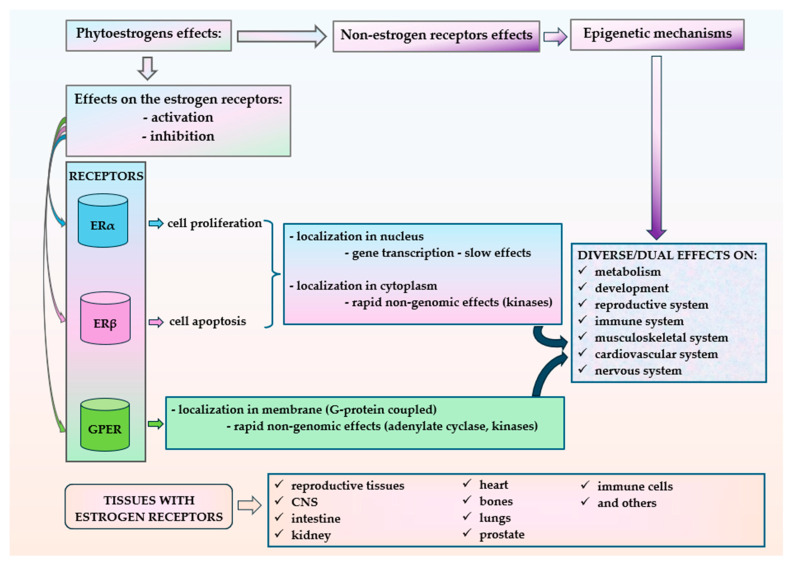
Principal effects of phytoestrogens on tissues.

Phytoestrogens can have both estrogenic and antiestrogenic effects, depending on their concentration, endogenous estrogen levels, target tissue, and factors such as sex or menopausal status [101]. They can also influence cellular processes through epigenetic mechanisms independent of estrogen receptor (Figure 5) [1]. Thanks to the structural similarity between phytoestrogens and endogenous steroidal estrogens in humans, they may act similarly by binding to estrogen receptors (ER). There are two types of ERs: estrogen receptor alpha (ERα) and beta (ERβ) [6,94,102]. ERα and ERβ are widely expressed in mammalian tissues but exhibit distinct distributions. ERα is most abundant in the uterus and is also present in reproductive organs, bones, and the hypothalamus, whereas ERβ is highly expressed in ovarian granulosa cells and the gastrointestinal tract, with lower levels in several reproductive and endocrine tissues [103]. These receptors have different functions: alpha receptors are involved in cell proliferation, while beta receptors play a key role in cell apoptosis [104]. After binding to a ligand, the receptor translocates from the cytoplasm to the nucleus, where it influences DNA transcription or small RNA, thereby affecting the expression of specific genes. Thus, phytoestrogens may potentially regulate all processes influenced by estrogen [6]. Phytoestrogens interact differently with the estrogen receptor subtypes ERα and ERβ due to variations in their chemical structures, which consequently influence their biological activity. Among them, coumarin-derived compounds, including coumestrol and psoralen, tend to preferentially interact with ERβ, suggesting potential relevance in tissues where this receptor subtype is more abundant, such as bone and cardiovascular tissues. Similarly, isoflavones like genistein and daidzein are capable of binding to both receptor subtypes, although they also exhibit greater affinity toward ERβ. The interaction between phytoestrogens and estrogen receptors is stabilized through hydrogen bonding and hydrophobic forces, contributing to tissue-specific responses that depend on receptor distribution. Since ERα is predominantly associated with reproductive tissues and cellular proliferation, while ERβ is more highly expressed in the brain and skeletal system and is linked to antiproliferative and pro-apoptotic effects, selective receptor modulation plays an important role in determining the physiological and therapeutic effects of phytoestrogens [105].

Recent studies indicate that phytoestrogens interact not only with the classical estrogen receptors ERα and ERβ but also exhibit specific binding affinity for the G protein-coupled estrogen receptor 1 (GPER1), formerly known as GPR-30 [106]. GPER (formerly GPR-30) belongs to the rhodopsin family and can activate different signaling pathways depending on ligand concentration, including a cAMP-dependent pathway and a Src/EGFR-mediated pathway. Because estrogen receptors vary in affinity, cellular location, activation mechanisms, and tissue distribution, the effects of estradiol and estrogen-like compounds are complex and often nonlinear, with some xenoestrogens showing bell-shaped or U-shaped dose–response curves both in vitro and in vivo [4,106]. Independent of their interactions with estrogen receptors, phytoestrogens can modulate several intracellular signaling pathways. They can activate insulin-like growth factor-1 and serotonergic receptors, influencing tyrosine kinase activity, as well as cyclic adenosine monophosphate (cAMP) activity, and modulate phosphatidylinositol-3 kinase/Akt and mitogen-activated protein kinases (MAPKs) pathways. Additionally, they can suppress nuclear factor kappa β (NF-κB)-dependent transcription, modify histones, and alter RNA expression, thereby acting as intracellular regulators of the cell cycle and apoptosis. Through these mechanisms, phytoestrogens exhibit antioxidant, antiproliferative, antimutagenic, and antiangiogenic effects that may contribute to improved health and longevity, and can regulate processes such as cell proliferation, apoptosis, inflammation, oxidative stress, and metabolic homeostasis, thereby contributing to potential cardioprotective, neuroprotective, anti-inflammatory, and anticancer effects. However, their overall impact is context-dependent and influenced by dose, tissue type, hormonal status, and duration of exposure, as these same pathways may also be involved in tumor progression and hormone-sensitive diseases [6,94,107].

### 4.3. Phytoestrogen Effect on Human Health

Phytoestrogens have been shown to exert a broad spectrum of potentially beneficial effects in humans across different sexes and age groups, including children. The biological impact of dietary phytoestrogens is multifactorial. It depends on factors such as the type and food source of the phytoestrogen, its concentration and bioavailability, as well as individual characteristics like ethnicity, hormonal status (related to age, sex, and physiological condition), and overall health status [7]. Their activity primarily aims to reduce the risk of post-menopausal symptoms. However, phytoestrogens have also demonstrated beneficial effects in various diseases, including osteoporosis, cardiovascular disease, metabolic syndrome, and type 2 diabetes [1]. They are an important group of molecules in treating different types of cancer, such as breast cancer in women [89], as well as some hormone-sensitive cancers and colorectal neoproliferative lesions [94]. These qualities make phytoestrogens promising candidates for improving human health. Nonetheless, their biological effects vary among individuals. Most studies on the relationship between phytoestrogens and human health have focused on soya-based phytoestrogens, which contain high levels of isoflavones. It has been noted that the traditional Asian diet contains significantly more soya products than the Western diet, resulting in higher urinary and plasma isoflavone levels in Asian populations than in Western populations [103]. In contrast, the most common dietary phytoestrogens in Western diets are flavanol and lignan compounds [108]. These differences have been associated with lower incidence rates of hormone-dependent diseases, particularly breast and prostate cancers, as well as reduced prevalence of menopausal symptoms, osteoporosis, and certain cardiovascular disorders in Asian populations compared with Western populations. However, these associations are influenced by multiple factors, including genetics, gut microbiota composition, lifestyle, and overall dietary patterns, making it difficult to attribute health outcomes exclusively to phytoestrogen intake [109].

#### 4.3.1. Effect of Phytoestrogens During Human Development and Puberty

Phytoestrogens can affect hormonal activity as early as infancy [108]. The fetus is exposed to phytoestrogens as they cross the placenta. After birth, breastfed infants may be exposed to phytoestrogens through maternal milk. However, most phytoestrogen intake occurs through direct consumption of soya products [103]. Still, no evidence suggests that they alter sex hormone levels in newborns [7]. Reports have indicated a higher rate of premature thelarche among infants fed soya-based diets. Additionally, the estrogen-like effects of soya products in infants are linked to the development and maturation of vaginal epithelial cells. These cells show a peak maturation index at birth due to maternal estrogen exposure, followed by a rapid decline in estrogenic influence by one month of age. This decreased maturation index persists in infants fed breast milk or cow’s milk; in contrast, infants consuming soya-based foods show a renewed increase in the vaginal maturation index by six months of age, indicating the estrogenic effects of soya products [110]. Few studies have explored the relationship between phytoestrogen intake and sexual development in children. One study examined whether soya intake affects sex steroid levels in girls (694 girls aged 3–24 months) and found that breast tissue in infants fed soya formula was more enlarged in the second year of life than in those who were breastfed or formula-fed [111]. A cross-sectional analysis of more than 400 preschool-aged children (ages 3–6), including boys and girls, examined the association between soya intake and urinary sex hormone levels in healthy participants. The results showed that higher soya (approximately 54–61 g/day) and soya isoflavone (approximately 24–27 mg/day) intake was associated with increased androgen levels in girls, while boys with higher soya intake had lower estrogen levels. One explanation is that soya components may inhibit aromatase activity, an enzyme that converts androgens into estrogens. This inhibition could lead to lower estrogen levels in boys and relatively higher androgen levels in girls with more soya in their diets. Overall, the findings suggest that dietary soya may influence the regulation or metabolism of sex steroids during early childhood, with sex-specific effects [112]. Another study evaluated the potential effect of soya intake on urinary sex steroid levels in a small group of girls (20 participants) aged 8–14 years. In this study, following a high-soya diet (daily serving of soy foods, including soymilk, soy nuts, or tofu, providing approximately 30 mg/day of isoflavones) for eight weeks resulted in modest, non-significant increases in total androgen and estrogen levels, along with insignificant decreases in pregnanediol [113]. Phytoestrogens, as exogenous factors, may influence the development of pubertal characteristics, as they belong to the group of endogenous disruptors. Because estradiol, LH, and FSH concentrations are inherently low before puberty, phytoestrogens may exert their effects without detectable alterations in circulating hormone levels, instead acting on estrogen-responsive tissues. [114]. Research presents conflicting evidence about the onset of puberty. Even a retrospective cohort study found no statistically significant differences between groups, in females or males, regarding self-reported pubertal development. According to this study’s results, there are no meaningful differences in overall health status or reproductive function between those exposed to soya and cow’s milk [115]. In another self-reported cohort study of women, following a soya diet was associated with both very early (≤10 years) and late (≥15 years) age at menarche [116]. Studies exploring the effects of phytoestrogens on pubertal timing indicate that higher prepubertal isoflavone intake in girls is linked to later puberty and delayed breast development [117,118,119]. Additionally, later puberty timing was observed in a study of girls and boys (aged 6–8 years), where increased soya intake was connected to delayed puberty [120]. In contrast, recent findings suggest a potential role for phytoestrogens in promoting earlier pubertal development. A prospective longitudinal study found that girls who were fed soya-based formula in early infancy experienced menarche earlier than those who were breastfed or received other types of formula. These findings further indicate that early-life exposure to soya products may increase the likelihood of menarche in early adolescence [121]. In addition, another study demonstrated an association between elevated serum isoflavone concentrations and central precocious puberty in girls (aged 8.6 ± 0.8 years) [122]. Endocrine-disrupting compounds, including phytoestrogens, have attracted increasing scientific attention due to their potential to interfere with hormonal regulation during critical periods of development. Such interference may alter normal developmental pathways, potentially leading to adverse effects on reproductive function later in life. Furthermore, these alterations may have long-term consequences extending into subsequent generations [123].

#### 4.3.2. Effect of Phytoestrogens on Fertility

Regarding phytoestrogen intake and its effects on human fertility, the underlying mechanisms are still not fully understood. The evidence remains inconclusive and mostly depends on the type and amount of phytoestrogens consumed. Most beneficial or neutral effects are associated with typical dietary levels rather than high pharmacological doses. Additionally, individual differences in how people metabolize phytoestrogens—especially their ability to produce equol—may significantly influence their biological effects. The link between phytoestrogen exposure and menstrual cycle features was studied in a group of healthy women (ages 18–40) trying to conceive. The findings showed no significant association between phytoestrogen intake and overall cycle length; however, some lignans appeared to be associated with better cycle regularity [124]. Phytoestrogen consumption did not strongly relate to fecundability in healthy women attempting to conceive [125]. A prospective cohort study exploring the impact of phytoestrogens on IVF outcomes in women (aged 18–42) suggests that dietary phytoestrogens could positively influence reproductive results for women undergoing IVF treatment. Higher urinary phytoestrogen concentrations were significantly associated with improved oocyte maturation and fertilization capacity, as well as an increased likelihood of clinical pregnancy and live birth. Furthermore, higher phytoestrogen levels in both follicular fluid and urine were associated with a greater likelihood of achieving a live birth per IVF cycle [126]. In recent decades, a decline in human sperm quality has been reported, potentially linked to increased exposure to environmental endocrine-disrupting compounds, including phytoestrogens. In a study examining the short-term effects of phytoestrogen supplementation on sperm quality and serum levels of sex steroids and gonadotropins in men, healthy volunteers took a supplement containing 40 mg of isoflavones daily for 2 months. It was observed that phytoestrogen supplementation led to elevated plasma levels of genistein and daidzein. Still, no significant changes were detected in hormonal profiles, testicular volume, or semen parameters throughout the study. Overall, the administered phytoestrogen dose did not negatively affect sperm quality [127]. On the other hand, some studies suggest that greater consumption of soya-based foods and soya isoflavones may be associated with reduced sperm concentration. Men with an average isoflavone intake of approximately 5.4 mg/day (higher soy intake) showed lower sperm concentration, with an average reduction of 35 million sperm/mL compared with non-consumers [128], and higher exposure to soya isoflavones, especially daidzein and genistein, was linked to an increased likelihood of infertility, particularly in men with abnormal semen parameters in a study involving Chinese men. The geometric means of urinary genistein and daidzein concentrations were 56.11 μg per g creatinine and 64.16 μg per g creatinine in idiopathic infertile men with at least one abnormal semen parameter [129]. Another study evaluated urinary isoflavone concentrations and semen parameters and observed negative associations between urine isoflavone concentration with sperm count and concentration, but not with sperm motility. The geometric mean of urinary genistein and daidzein concentrations were 538 μg per g creatinine and 1178 μg per g creatinine, respectively, in Japanese men [130].

#### 4.3.3. Phytoestrogens and Their Use for Menopausal Symptoms

The climacteric is a transitional stage in a woman’s life characterized by significant hormonal shifts and physical, psychological, and social changes. It encompasses two main phases: perimenopause, which begins with the first symptoms and ends at the final menstrual period, and postmenopause, which continues into later adulthood, usually up to the mid-sixties. The intensity and type of menopausal symptoms vary widely among women. They can negatively impact self-esteem, health status, and overall quality of life, even though menopause is a natural biological process that typically occurs around age 51 [131]. Common symptoms include sleep disturbances, genitourinary discomfort, mood fluctuations, cognitive changes, decreased sexual desire, reduced vaginal lubrication, weight gain, bone loss, vasomotor issues (such as night sweats and hot flashes), and unfavorable alterations in metabolic health [131,132,133].

*Phytoestrogen and bone loss*

Estrogens play a vital role in maintaining bone mass by reducing osteoclast activity and supporting osteoblast function, thereby preserving bone structure. They also facilitate nutrient absorption, thereby contributing to overall bone health. When estrogen levels decrease, as in menopause, bone turnover increases, leading to greater resorption and a higher risk of osteoporosis. Hormone replacement therapy can help counteract this loss, but plant-derived compounds known as phytoestrogens may offer a natural alternative. By mimicking or modulating estrogen activity, phytoestrogens can help maintain bone density and potentially reduce the risk of osteoporosis [134,135]. The study aimed to evaluate the effect of isoflavone supplements on bone mineral density in postmenopausal women through a systematic review and meta-analysis. The results show that isoflavone treatments—especially those providing at least 50 mg/day of genistein—are effective in increasing bone mineral density in this group [135]. Other meta-analyses of randomized controlled trials indicate that daily intake of 40–300 mg of isoflavones (average 106 mg) for 6–24 months results in a moderate but statistically meaningful increase in bone mineral density compared to controls, suggesting that soya isoflavones can effectively slow postmenopausal bone loss [136].

*Phytoestrogens and vasomotor symptoms*

Vasomotor symptoms, such as hot flashes and night sweats, are common during the menopausal transition, affecting nearly 80% of women worldwide, and can vary in severity with noticeable impacts on quality of life and overall health [137,138]. The incidence of vasomotor symptoms during the climacteric period varies across populations, with phytoestrogen intake appearing to be an important factor [139]. Several studies have explored the effects of phytoestrogen formulations on vasomotor symptoms. Meta-analyses suggest that phytoestrogens may slightly reduce the frequency of hot flashes and vaginal dryness in menopausal women, while showing no significant effect on night sweats and no serious adverse effects [140,141].

Several reviews show inconclusive evidence regarding the effectiveness of phytoestrogens in reducing vasomotor symptoms; no consistent reductions in night sweats were observed, and mixed results for hot flashes were reported. Studies on lignans showed no significant effects, although possible synergy with isoflavones was suggested. Notably, some trials indicate that high-dose genistein may decrease hot flash frequency, suggesting it could be more effective than other phytoestrogens [133].

#### 4.3.4. Phytoestrogens in Cancer Prevention

Phytoestrogens have been extensively studied for their role in cancer prevention, although their impact on treatment outcomes remains debated. Some evidence suggests that they may impair the efficacy of anticancer therapies by modulating oxidative stress and other pathways. On the other hand, certain phytoestrogens could protect healthy cells, potentially reducing the side effects of cancer treatment [13]. Soya-based preparations have been proposed for the prevention and management of certain cancers. However, clinical evidence indicates that isoflavones, due to their estrogenic and proliferative properties, may increase breast cancer risk in susceptible individuals [66]. Activation of ERα can promote carcinogenesis, while activation of ERβ is associated with antiproliferative and anticarcinogenic properties. Importantly, the ER often exert opposing effects on apoptosis, migration, and proliferation, highlighting their distinct roles in cancer development [12,142]. Experimental studies show that phytoestrogens can modulate cell signaling pathways, affect the cell cycle, and induce apoptosis in breast cancer cells [143]. A meta-analysis of prospective studies found that every 10 mg/day increase in soya isoflavone intake was associated with a 3% reduction in breast cancer risk. The China Kadoorie Biobank study revealed that moderate soya consumption was not linked to breast cancer risk among Chinese women. However, these findings suggest that higher soya intake may provide potential benefits for breast cancer prevention [144]. A meta-analysis of prospective studies examining soya isoflavone intake and breast cancer risk showed a clear inverse relationship between isoflavone consumption and breast cancer incidence in both pre- and postmenopausal women. These results indicate that soya isoflavone intake may reduce breast cancer risk across these groups [145]. Plant lignans may be associated with a modest reduction in postmenopausal breast cancer risk. In a case–control study, higher dietary lignan intake was linked not only to reduced breast cancer risk but also to tumors with more favorable prognostic features [146,147]. A case–control study examined dietary phytoestrogen intake and the risk of ovarian (1366 cases) and endometrial (1288 cases) cancers. The study suggests a potential inverse association between phytoestrogen intake and ovarian cancer risk, with the effect mainly driven by mucinous tumors. Most findings were not statistically significant at the low intake levels observed (median 1.27 mg/day). No association was found for other ovarian subtypes or for endometrial cancer [148]. Furthermore, in a randomized controlled trial investigating the effects of phytoestrogens on prostate cancer, a diet high in phytoestrogens was found to reduce tumor proliferation in men with prostate cancer, and this effect may vary among men with different polymorphisms of the estrogen receptor beta (ERβ) gene. Therefore, a diet rich in phytoestrogens may lead to lower tumor proliferation and prostate-specific antigen concentrations in men with prostate cancer who have a specific ERβ polymorphism [149]. Another meta-analysis indicates that higher phytoestrogen intake, especially of genistein and daidzein, may be linked to a lower risk of prostate cancer. Additionally, elevated serum levels of enterolactone were associated with a significant reduction in prostate cancer risk [150]. Table 2 summarizes the potential anticancer effects of selected phytoestrogens.

#### 4.3.5. Phytoestrogens—Glucose Homeostasis and Obesity

Phytoestrogens, particularly isoflavones such as genistein and daidzein, modulate glucose homeostasis and obesity through a complex interplay of estrogen-dependent and estrogen-independent mechanisms. Although preclinical studies have consistently demonstrated their protective effects, findings from human clinical trials remain heterogeneous, varying according to the specific phytoestrogen administered, dosage, sex of the participants, and menopausal status [177].

Phytoestrogens regulate glucose metabolism through multiple biochemical pathways. Isoflavones such as genistein function as α-glucosidase inhibitors, thereby slowing carbohydrate digestion and absorption in the small intestine and consequently reducing postprandial glucose excursions [178]. In addition, phytoestrogens suppress hepatic expression of phosphoenolpyruvate carboxykinase (PEPCK) and glucose-6-phosphatase (G6Pase), two key enzymes involved in gluconeogenesis [179]. Inhibition of these enzymes contributes to improved glycemic control under hyperglycemic conditions [1]. Furthermore, daidzein and its metabolite equol have been shown to upregulate the expression of glucose transporter type 4 (GLUT4) and insulin receptor substrate-1 (IRS-1), thereby enhancing glucose uptake in peripheral tissues, including skeletal muscle and adipose tissue [179]. Daidzein has also been reported to increase hepatic glucokinase (GK) activity, promoting intracellular glucose utilization and energy metabolism [178].

Phytoestrogens exert anti-obesity effects through modulation of adipose tissue distribution and lipid metabolism. In particular, isoflavones have been shown to reduce circulating levels of low-density lipoprotein (LDL) cholesterol and triglycerides while increasing high-density lipoprotein (HDL) cholesterol concentrations [180]. Genistein further inhibits adipogenesis and suppresses adipocyte proliferation and differentiation through downregulation of genes involved in lipogenesis [180]. Moreover, a high dietary intake of lignans has been inversely associated with body mass index and waist-to-hip ratio in postmenopausal women [181].

The effects of phytoestrogens on insulin metabolism are highly dependent on the specific compound administered. Meta-analyses have demonstrated that isolated genistein significantly reduces fasting insulin concentrations and improves the homeostatic model assessment of insulin resistance (HOMA-IR) in postmenopausal women with metabolic syndrome or type 2 diabetes mellitus [177]. In addition, genistein acts as a survival factor for pancreatic β-cells by protecting them against apoptosis and enhancing insulin secretion via cyclic adenosine monophosphate (cAMP)-mediated signaling pathways [178]. Phytoestrogens have also been shown to activate AMP-activated protein kinase (AMPK) in skeletal muscle, a mechanism analogous to that of metformin, thereby promoting insulin sensitivity [182]. However, although isolated phytoestrogen compounds frequently demonstrate beneficial metabolic effects, mixtures of isoflavones have in some studies been associated with an unfavorable glycemic profile, including increased insulin concentrations and elevated HOMA-IR values [177].

Epidemiological and clinical studies suggest that phytoestrogens may exert a protective effect against type 2 diabetes mellitus (T2D), particularly in women. Higher dietary intake of phytoestrogens has been associated with an approximately 10% reduction in T2D risk among female populations, with the strongest associations observed for soy-derived isoflavones and soy-based products [177]. In addition, genistein may confer protection against diabetic complications, including neuropathy, nephropathy, and retinopathy, through its antioxidative and anti-inflammatory properties [182,183].

The daidzein-derived metabolite S-equol, produced by intestinal microbiota, exhibits substantially greater biological activity than its precursor compound. Individuals capable of producing equol (“equol producers”), owing to a specific gut microbial composition that enables the conversion of daidzein to equol, may therefore derive greater antidiabetic benefits from soy consumption than non-producers; however, this metabolic capability is present in only approximately 30–50% of the population [4,184]. Notably, the beneficial association between phytoestrogen intake and T2D risk appears to be more pronounced in women, particularly postmenopausal women with lower endogenous estrogen levels, whereas studies conducted in men have generally failed to demonstrate a significant association between isoflavone intake and T2D risk [185].

#### 4.3.6. Phytoestrogens and PCOS

Isoflavones like genistein and daidzein are structurally and functionally similar to mammalian estrogens. Because of this similarity, they can act as either estrogen agonists or antagonists, depending on their concentration and the level of endogenous estrogen in the body. In the context of Polycystic Ovary Syndrome (PCOS)—a complex endocrine and metabolic disorder—phytoestrogens are associated with several potential therapeutic benefits, primarily by addressing hyperandrogenism, insulin resistance, and oxidative stress [186]. More recently, PCOS has been proposed to be renamed as “polyendocrine metabolic ovarian syndrome” in an effort to better reflect the multifactorial and systemic nature of the condition. However, as this nomenclature transition remains ongoing and has not yet been universally adopted, the traditional term PCOS will be used throughout this text [187].

Phytoestrogens influence the hormonal milieu of PCOS through several interconnected pathways. The most significant hormonal benefit is the reduction in testosterone levels [186]. This effect is mediated, at least in part, through inhibition of key steroidogenic enzymes, including 3β-hydroxysteroid dehydrogenase (3β-HSD) and 17β-hydroxysteroid dehydrogenase (17β-HSD), both of which play essential roles in androgen biosynthesis [188]. PCOS is frequently characterized by elevated LH concentrations and an increased LH/FSH ratio [188]. Administration of phytoestrogens has been shown to significantly reduce serum LH levels and normalize the LH/FSH ratio in both human and experimental animal studies [189]. Furthermore, in rat models of PCOS, isoflavones have demonstrated restorative effects on ovarian morphology and function, including reduction in the number of cystic (atretic) follicles, increased thickness of the granulosa cell layer, and higher numbers of healthy follicles and corpora lutea [190].

In experimental rat models of PCOS, isoflavones have been shown to restore normal ovarian structure and function. Their administration is associated with a reduction in the number of cystic follicles, increased thickness of the granulosa cell layer, and a higher number of healthy follicles and corpora lutea [190]. In addition, some studies suggest that phytoestrogens may increase circulating levels of sex hormone-binding globulin (SHBG), thereby reducing the biological availability of free testosterone. However, clinical evidence in humans regarding this specific effect remains inconclusive [188,191]. Phytoestrogens may also ameliorate the metabolic disturbances associated with PCOS, including obesity, dyslipidemia, and impaired glucose metabolism, as discussed previously. PCOS is increasingly recognized as a state of chronic low-grade inflammation accompanied by enhanced oxidative stress. Phytoestrogens appear to attenuate these pathological processes through modulation of inflammatory and antioxidative pathways [186]. Specifically, they have been shown to reduce circulating levels of pro-inflammatory cytokines, including tumor necrosis factor-α (TNF-α) and interleukin-6 (IL-6), both of which are typically elevated in PCOS and contribute to the development of hyperandrogenism and insulin resistance [186]. Phytoestrogens increase the activity of antioxidant enzymes such as superoxide dismutase (SOD), glutathione peroxidase (GPx), and catalase, while reducing markers of lipid peroxidation like malondialdehyde (MDA) [189].

## 5. Conclusions

Phytoestrogens are a diverse class of plant-derived compounds with a structural similarity to estradiol and the ability to interact with estrogen-related signaling pathways. However, their biological effects are heterogeneous and depend on the class of compounds, the level of exposure, and population characteristics, with considerable interindividual variability in metabolism and response. Current research suggests that these compounds confer health benefits at different stages of life, as well as in various diseases. While isoflavones are the most extensively studied class of phytoestrogens, evidence for other classes of phytoestrogens remains more limited and less consistent. Although current findings are promising, integrated clinical and mechanistic research is still needed. Therefore, future studies could focus on elucidating the mechanisms related to estrogen receptors, bioavailability, and clinical relevance of known as well as less-studied phytoestrogen candidates.

## Figures and Tables

**Table 1:** Summary of phytoestrogen classification, their dietary sources and health effects.

***I.*** ***Phenolic acids*** *(contain a phenolic ring and a carboxylic acid group)*	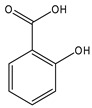	
*Example*	*Dietary Source*	*Health Effect*
Caffeic acid (3,4-dihydroxycinnamic acid)	lingonberries, black chokeberries, apples, plums, red wine, coffee, herbs of the mint family (e.g., sage, oregano, thyme, marjoram) [14,15]	antioxidant, anti-inflammatory, anticancer, antihyperglycemic [16]
Gallic acid	guava, mango, grapes, pomegranate, avocado, blackcurrant [14,17]	antioxidant, anti-inflammatory, antineoplastic, therapeutic activities in gastrointestinal, neuropsychological, metabolic, and cardiovascular disorders [18]
***II.*** ***Flavonoids***		
**Prenylated Flavonoids**	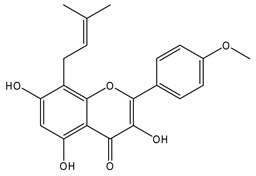	
*Example*	*Dietary Source*	*Health Effect*
8-Prenylnaringenin	hop, beer [19,20]	menopausal and post-menopausal symptoms treatment, prevention of bone-resorption, inhibition of tumor growth [21]
Isoxanthohumol	beer, hop [22,23]	antioxidant, anticancer, estrogenic, anti-inflammatory [24]
**Flavones**	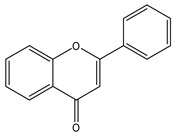	
*Example*	*Dietary Source*	*Health Effect*
Luteolin	carrots, peppers, celery, pomegranates, and herbs such as peppermint, rosemary, and parsley [25,26]	antiallergic, anti-inflammatory, antidiabetic, neuroprotective, and anticancer, antioxidant [27]
Apigenin	parsley, celery, peppermint, thyme, chamomile [25,26]	antibacterial, antiviral, antiproliferative, anti-inflammatory, antioxidant, antiangiogenic, anticancer activities [28]
**Isoflavones**	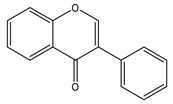	
*Example*	*Dietary Source*	*Health Effect*
Genistein	soybeans, soya products, legumes, alfalfa, red clover, white clover [19,22,29,30,31,32]	antioxidant, anti-inflammatory, antibacterial, antiviral, angiogenesis-modulating effect, estrogen-like activity [33]
Daidzein	soybeans, soya products, red clover [22,31,32]	antioxidant, antidiabetic, anti-inflammatory, nephroprotective, neuroprotective, anticancer, antihyperlipidemic effects, estrogen-like activity [34]
Formononetin	soybeans, soya products, red clover [32,35,36]	antioxidant, anticancer, anti-inflammatory [37]
Biochanin A	chickpea, red clover, soybean [19,38]	antioxidant, anticancer, anti-inflammatory, hypoglycemic [38]
Equol—produced from daidzein	soya products [14]	antioxidant, estrogen-like activity, anticancer, antibacterial [39]
**Flavonols**	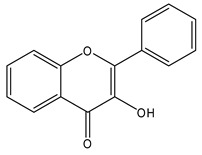	
*Example*	*Dietary Source*	*Health Effect*
Kaempferol	apples, blueberries, cherries, cranberries, broccoli, asparagus, kale, lettuce, onions, spinach, chives, dill, fennel leaves, Chinese cabbage [25,32,40]	antioxidant, anti-inflammatory, cardioprotective, neuroprotective, hepatoprotective, antidiabetic, antitumor [41]
Quercetin	apples, blueberries, cherries, cranberries, broccoli, asparagus, kale, lettuce, onions, spinach, chives, dill, fennel leaves, oregano, chili pepper [25,32,40]	antioxidant, anti-inflammatory, antiviral, anticancer [42]
**Flavanols**	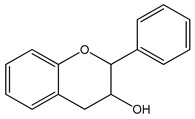	
*Example*	*Dietary Source*	*Health Effect*
Catechin	tea, cocoa, fruits (e.g., grape, peach, nectarine, plum), nuts [25,32,43]	antioxidant, antibacterial, anticataract, anti-inflammatory, anticancer [44]
Epicatechin	tea, cocoa, fruits (e.g., blackberry, grape, apricot, peach, sweet cherry) [25,32,43]	antioxidant, antimicrobial, anti-inflammatory, antitumor, cardioprotective [45]
**Flavanones**	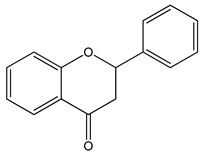	
*Example*	*Dietary Source*	*Health Effect*
Naringenin	citrus fruits, rosemary, red wine, grapefruit juice, orange juice [25,46,47]	antidiabetic, anticancer, antimicrobial, antiobesity, gastroprotective, immunomodulator, cardioprotective, nephroprotective, neuroprotective [48]
Hesperetin	lemons, limes, tangerines, grapes [25,49]	anti-inflammatory, antioxidant, antitumor, antibacterial [50]
**Coumestans**	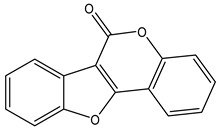	
*Example*	*Dietary Source*	*Health Effect*
Coumestrol	spinach, Brussels sprouts, soya beans, legumes, clover, kala chana (a type of chickpea), alfalfa sprouts, lima beans, pinto beans, split peas [19,22,51]	antioxidant, antimicrobial, antiviral, antimutagenic, anticancer, antiobesity, anti-inflammatory, estrogen-like activity [52,53,54]
Plicadin	split peas, pinto and lima beans, spinach, broccoli, Brussels sprouts, soybean sprouts [22,55]	estrogenic activity [7]
Repensol	split peas, pinto beans, lima beans, alfalfa, clover sprouts [56]	estrogenic activity [56]
Trifoliol	split peas, pinto beans, lima beans, alfalfa, clover sprouts [56]	estrogenic activity [56]
**Chalcones**	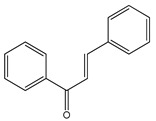	
*Example*	*Dietary Source*	*Health Effect*
Arbutin	Japanese pear trees, bearberries, pears, blueberries, cranberries, and marjoram [25,32,57]	antioxidant, antimicrobial, anti-inflammatory, anticancer [58]
Butein	dahlia, butea, and coreopsis plants, pod vegetables [25,32,59]	antioxidant, anti-inflammatory, anticancer, antidiabetic, hypotensive, neuroprotective properties [60]
***III.*** ***Non-Flavonoids***		
**Lignans**	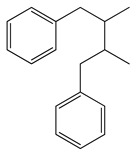	
*Example*	*Dietary Source*	*Health Effect*
Sesamin	sesame seeds [22,61]	antioxidant, antihypertensive, hypolipidemic, antiproliferative effects [62]
Matairesinol	flaxseed (linseed), grains, broccoli, cabbage, Brussels sprouts, pears, grapefruits, olives [22,63]	anti-inflammatory, anticancer, antiangiogenic properties [64]
Secoisolariciresinol	soybeans, flaxseed, grains, broccoli, cabbage, Brussels sprouts, pears, grapefruits, olives, cocoa powder, plain chocolate [19,63]	anticancer, antioxidant, cardiovascular disease risk reduction [65]
Enterodiol	flaxseeds, whole grains, fruits and vegetables, sesame seeds, legumes [14,25,66]	antioxidant, estrogenic activity, cardiometabolic protective effects [67]
Enterolactone	flaxseeds, whole grains, fruits and vegetables, sesame seeds, legumes [14,25,66]	antioxidant, estrogenic activity, cardiometabolic protective effects [67]
**Stilbenes**	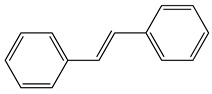	
*Example*	*Dietary Source*	*Health Effect*
Resveratrol	grapes, wine, peanuts, mulberries, blueberries, strawberries [14,25,68]	antioxidant, anti-inflammatory, antiobesity, anticancer, anti-diabetic, cardiovascular, and neuroprotective properties [69]
**Saponins**	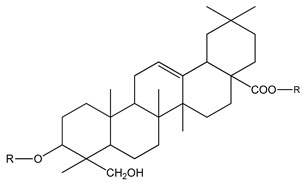 R = sugar moiety	
*Example*	*Dietary Source*	*Health Effect*
Ginsenosides (dioscin, diosgenin, gypenoside XVII, protopanaxadiol/protopanaxatriol)	American ginseng, Asian ginseng [70,71]	anticancer, antidiabetic, antioxidant, neuroprotective and cardioprotective effects, immunomodulatory effects [72]
**Terpenes/Terpenoids**	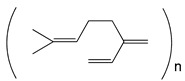	
*Example*	*Dietary Source*	*Health Effect*
Retinoids (vitamin A/retinol, retinal, retinoic acid, and their derivatives)	egg yolks, broccoli, spinach, avocado, sweet potatoes, whole grains, cereal, dry nuts [70,73]	antioxidant, antiwrinkle, anticomedogenic, antitumor, immunomodulatory effects [74]
Vinca alkaloids (vinblastine, vincristine, vinorelbine)	periwinkle plant [70,75]	antidiabetic, antihypertensive, anticancer, cytotoxic, hypoglycemic, cytotoxic effects [76]
Carotenoids (lycopene, β-carotene, phytoene, phytofluene)	carrots, apricots, pumpkins, red pepper, celery, tomatoes, apples, oranges, cherries [70,77]	antioxidant, anti-inflammatory, anticancer, immunomodulatory effect, anticancer, visual health protection, cardioprotective effects [78]
Tocopherols	almond oil, other nut oils, sunflower oil, corn oil, olive oil, rapeseed oil, linseed oil, soybean oil [70,79]	antioxidant, anti-inflammatory, antiatherosclerotic, anticancer, antiallergic, antilipidemic, antidiabetic, antihypertensive, antiobesity, immunomodulatory and cardioprotective effects [79]
Tocotrienols (members of the vitamin E family)	olive oil, sunflower oil, flaxseed oil, poppy seed oil, grapefruit seed oil, wheat germ, barley, oats, hazelnuts, maize, buckthorn berry, rye [70,79,80]	antioxidant, anti-inflammatory, antiatherosclerotic, anticancer, antiallergic, antilipidemic, antidiabetic, antihypertensive, antiobesity, immunomodulatory and cardioprotective effects [79]
Meroterpenes (terpenophenolics)	fungi, marine organisms, plants from the genera psidium, eucalyptus, arnebia, eugenia [70,81]	anticancer, cytotoxic [82]
Citral	lemon grass [70,83]	antioxidant, antifungal, antibacterial, anti-inflammatory [84]
Eugenol	tulsi, pepper, clove, cinnamon, cinnamon bark and leaves, turmeric, ginger, oregano, thyme, basil, bay, marjoram, mace, nutmeg [70,85]	antibacterial, antiviral, antifungal, anticancer, anti-inflammatory, antioxidant [86]
Geraniol	lavender, lemongrass, citronella, essential oils of fruits, and vegetables [70,87]	antioxidant, antimicrobial, antitumor, anti-inflammatory [88]

**Table 2:** Anticancer potential of selected phytoestrogens.

Phytoestrogen	Cancer Type	Effect	Reference
Genistein	Breast cancer	Angiogenesis inhibition via VEGF and HIF-1α pathways	[151,152]
		Inhibition of breast cancer cell proliferation	[153]
		Regulation of Bcl-2 and MEK5/ERK5; apoptosis induction; growth inhibition	[154]
	Prostate cancer	Induces apoptosis by inhibiting STAT3, AKT, ERK, and p38	[155]
	Endometrial cancer	Endometrial cancer cell proliferation inhibition via cell cycle arrest	[156]
		Endometrial cancer growth inhibition via G2 arrest and apoptosis; modulation of ERα, hTERT, PR, AKT/mTOR, and MAPK pathways	[157]
Daidzein	Breast cancer	MCF-7 growth inhibition; ROS-mediated mitochondrial apoptosis (downregulation Bcl-2, upregulation Bax, cytochrome c release, caspase-9/-7 activation	[158].
	Endometrial cancer	Tissue-selective estrogenic/anti-estrogenic effects (ERα vs. ERβ); ERα upregulation and nuclear localization; reduced proliferation, migration and invasion; cell cycle arrest and apoptosis induction	[159,160]
Coumestrol	Breast cancer	decrease ERα protein/mRNA levels; inhibition of cell viability, growth and proliferation; upregulation Bax, apoptosis, cell cycle arrest, ROS generation, DNA damage, ERK1/2 phosphorylation and p53; inhibition AKT phosphorylation	[161,162]
	Prostate cancer	Inhibition of cell proliferation and migration; induction of apoptosis; ROS generation; DNA damage; mitochondrial dysfunction; cell cycle arrest; modulation of PI3K/AKT signaling and activation of ERK1/2 and JNK pathways	[163]
Secoisolariciresinol	Breast cancer	Induction of PARP cleavage and downregulation of ERα expression	[164]
Sesamin	Colorectal cancer	Downregulation of IκBα and p65; HIF-1α and VEGFA suppression	[165]
Matairesinol, secoisolariciresinol	Colorectal cancer	Downregulation of ULK1/2	[166].
Kaempferol	Endometrial cancer	Induction of apoptosis; sub-G1 cell cycle arrest; suppression of ERα and survivin expression; downregulation of Bcl-2; inhibition of estrogen-induced ERα–survivin signaling	[167]
	Ovarian cancer	Inhibition of angiogenesis and tumor growth; downregulation of VEGF expression; suppression of HIF-1α and ESRRA; inhibition of AKT phosphorylation; modulation of HIF-dependent (AKT/HIF-1α) and HIF-independent VEGF signaling pathways	[168]
		Induction of intrinsic apoptosis (caspase-3/7 activation); upregulation of p53, Bad, and Bax; downregulation of Bcl-xL; activation of mitochondrial (intrinsic) apoptotic pathway	[169]
	Pancreatic cancer	Induction of apoptosis; suppression of cell migration; inhibition of EGFR-mediated signaling pathways (Src, ERK1/2, AKT)	[170]
		Induction of apoptosis via ROS generation; suppression of Akt/mTOR signaling; downregulation of TGM2 expression; TGM2-mediated modulation of ROS-dependent apoptotic pathway	[171].
Quercetin	Breast cancer	Downregulation of MMP-2, MMP-9 and VEGF; induction of autophagy; inactivation of Akt/mTOR signaling	[172]
		Inhibition of PI3K/AKT/mTOR signaling	[173]
	Prostate cancer	Inhibition of PI3K/AKT/ERK1/2 signaling	[174]
Resveratrol	Breast cancer	Downregulation of NF-κB, COX-2, and MMP-9	[175]
	Prostate cancer	Upregulation of ERβ and IGF-I; downregulation of phospho-ERK1 and ERK2	[176]

## Data Availability

Not applicable.
